# Effect of Temperature on the Cyclic Fatigue Resistance and Phase Transformation Behavior of Three Different NiTi Endodontic Instruments

**DOI:** 10.7759/cureus.52916

**Published:** 2024-01-25

**Authors:** Esra İrem Yi̇ği̇t, İrem Çetinkaya

**Affiliations:** 1 Dentistry, Private Clinic, Istanbul, TUR; 2 Endodontics, Trakya University, Edirne, TUR

**Keywords:** nickel titanium, martensite, austenite, dsc, cyclic fatigue

## Abstract

Background

This study aims to analyze the phase transformation behavior and evaluate the effect of different temperatures on the fracture strength of files.

Methodology

A total of 108 files were used, and cyclic fatigue tests were performed on HyFlex EDM, VDW.ROTATE, and TruNatomy at three different temperatures (+4°C, +35°C, and +60°C) The time to fracture of files was measured, and the number of cycles was calculated. In addition, the fractured fragment lengths were measured. The Kruskal-Wallis test was used to compare the data that were not normally distributed according to groups. The Mann-Whitney U test was performed for comparisons. P-values <0.05 were considered statistically significant.

Results

As the temperature increased for all files, the number of cycles to fracture (NCF) decreased. NCF was significantly higher for ROTATE at 4°C and 60°C and HyFlex EDM at 35°C.

Conclusions

The resistance to cyclic fatigue in all files decreased significantly as the temperature increased.

## Introduction

Chemomechanical shaping is one of the most important steps in root canal treatment [1]. The root canal preparation facilitates the removal of infected root dentin and pulp, as well as the disinfection of root canals with irrigation solutions and medicaments [2].

Nickel-titanium (NiTi) rotary files provide an advantage over traditional stainless steel files in root canal shaping, especially in canals with curvature [3]. Super flexibility, high fracture toughness, excellent shaping ability, cutting efficiency, and fatigue resistance are the outstanding features of NiTi endodontic files. However, despite these advantages, unexpected fractures may occur [4].

Two different mechanisms, torsional and cyclic fatigue fracture, can break the instruments. Breakage because of torsion occurs when the file continues to rotate, even though the tip of the file is stuck in the root canal dentine. Cyclic fatigue fracture is more common in curved canals because of the repeated compression and tensile stresses created while the instrument is rotating [5]. As the maximum tensile stress on the instrument surface, which occurs at the maximum bending point during the shaping of a curved root canal, increases, the cyclic fatigue resistance decreases, and the instruments break faster [6]. Cyclic fatigue is the main cause of fractured endodontic instruments at 44.3% [7].

Instruments in the austenite phase at high temperatures transform into the martensite phase upon cooling or stress application. Superelasticity occurs in a reversible phase transformation between austenite and martensite [8]. The transition phase temperatures of instruments can vary with the alloys and heat treatments during manufacture [9]. Clinically, heating of NiTi instruments may cause them to transition toward the more rigid austenite phase, which makes the instruments more vulnerable to fatigue crack propagation compared with a file in the martensite phase [10]. The increased ductility of a file in the martensite phase makes it highly elastic with better resistance to fatigue crack growth, providing a higher resistance to cyclic fatigue [10]. Therefore, transformation temperatures have a critical effect on the mechanical properties and behavior of NiTi. During root canal preparation, heating or cooling irrigation solutions may affect the martensite-austenite phase transition of NiTi files, affecting the cycles to fracture [9]. Although the heated sodium hypochlorite solution increases the disinfection efficiency [11], heating the solution causes NiTi instruments to transition to the austenite phase, which reduces flexibility and cyclic fatigue resistance [10]. Studies have shown that body temperature affects the cyclic fatigue resistance of NiTi instruments [9,12]. Therefore, performing a cyclic fatigue test at root canal temperature is important [12].

The TruNatomy (Dentsply Sirona, Ballaigue, Switzerland) has recently been developed as a specially designed novel heat-treated NiTi instrument. These files have an off-center parallelogram square cross-section design and a variable regressive taper for minimal invasive preparation. These files go through various heat treatments, increasing their resistance to fatigue [13].

The VDW.ROTATE (VDW, Munich, Germany) has an S-shaped cross-section, off-center design and a constant taper. These files are produced via a special heat treatment developed by the manufacturer [14].

The HyFlex EDM (Coltene/Whaledent, Altstätten, Switzerland) contains a controlled memory (CM) wire that optimizes the microstructure of the NiTi alloy; however, electric discharge processing technology is also used. HyFlex EDM OneFile (25/~) has a tip size of 25 and a taper of 0.08 in the apical portion with a variable taper, gradually decreasing to 0.04 in the coronal region. The HyFlex EDM file has a quadratic cross-section at the tip, a trapezoidal cross-section in the middle, and an almost triangular cross-section at the top [15].

This study aims to analyze the phase transformation behaviors of TruNatomy, VDW.ROTATE and HyFlex EDM rotary NiTi instruments and evaluate the effect of different temperatures on their cyclic fatigue resistance.

## Materials and methods

Sample preparation

The power calculation was performed using G*Power 3.1.2 (Heinrich Heine University, Dusseldorf, Germany) software to determine the sample size for each group. The calculation indicated that the sample size should be a minimum of 12 files. Three continuous rotary instruments, i.e., TruNatomy (size 26, 04 taper), VDW.ROTATE (size 25, 04 taper), and HyFlex EDM (size 25, variable taper), were used. All files were examined under ×20 magnification with light microscopy (Leica DM 6000B; Leica Microsystems, Wetzeler, Germany), and files with defects or irregularities on the surface were not included in the study. A total of 108 files were used in the study. Cyclic fatigue tests were performed on three different file brands at three temperatures (+4°C, +35°C, and +60°C).

Preparation of the artificial canal and assembly

The cyclic fatigue resistance was performed using a custom-made test device with the handpiece of the VDW Silver Reciproc (VDW Munich, Germany) motor attached to a water bath. A stainless steel artificial canal prepared in a previous study was used [16]. In the dynamic cyclic fatigue testing, the TA HD Plus Texture device (Stable Micro Systems, UK) and the endodontic motor were fixed at the same angle each time with the help of an apparatus, and a 3 mm/second up and down movement in the artificial canal was ensured as in previous studies [17]. In the dynamic cyclic fatigue resistance test, the length of the instrument was set to 16 mm with a 3-mm axial dynamic motion [17] to reach 19 mm. A metal block was mounted in a 2.5% sodium hypochlorite-filled water bath, which kept the temperature constant. During the experiments, the temperature of sodium hypochlorite was continuously controlled with a digital thermometer placed in the water bath.

The files from each brand were rotated at 4°C, 35°C, and 60°C, respectively, following the instructions of the manufacturer (TruNatomy, 500 rpm/1.5 Ncm; VDW.ROTATE, 400 rpm/2.3 Ncm; HyFlex EDM, 400 rpm/2.5 Ncm) until fracture occurred in the artificial canal. The time to fracture was recorded in seconds for each instrument using a digital chronometer and fracture time was confirmed with a slow motion camera. The number of cycles to fracture (NCF) for each file was calculated. A digital caliper was used to determine the length of the separated part. The length was measured to determine which region of the file accumulated the most stress in dynamic motion.

Differential scanning calorimetry analysis

The transition temperatures of the three different rotary file systems were analyzed using differential scanning calorimetry (DSC) (DSC1, Mettler Toledo, Schwerzenbach, Switzerland). Two segments of 3-4 mm, between 10 and 15 mg of weight, were prepared from each instrument using a side cutter plier. The samples were placed in an aluminum pot. Analysis was performed in an atmosphere of argon gas, with temperatures ranging between −80 and 80°C with linear heating and cooling rates of 10°C/minute. Liquid nitrogen was used for cooling. The measurements were repeated three times for each sample. Phase transformation temperatures were determined from the intersection of the maximum gradient of the curve and baseline. The martensitic transformation-starting and finishing points (MS, MF) and austenite transformation-starting and finishing points (AS, AF) were determined using the software STARe Evaluation Software (Mettler Toledo) according to a previously reported study [18].

Scanning electron microscopy analysis

All files were analyzed using scanning electron microscopy (SEM) to determine the topographic features. First, the files were cleaned in an ultrasonic bath for three minutes using alcohol. The samples were placed in SEM (ZEISS EVO Scanning Electron Microscope, Oberkochen, Germany). The images were taken from the broken file surfaces under different magnifications (×400 and ×1,000). SEM photographs were analyzed by fractographic analysis for fracture origins and fatigue lines to confirm that the fracture was caused by cyclic fatigue.

Statistical analysis

Data were analyzed using SPSS version 23 (IBM Corp., Armonk, NY, USA). The normal distribution was evaluated using the Shapiro-Wilk test. The Kruskal-Wallis test was used to compare non-normally distributed data. The Mann-Whitney U test was performed to compare the groups. P-values <0.05 were considered significant.

## Results

All NiTi rotary files used in our study were used at three different temperatures (4°C, 35°C, and 60°C) according to the manufacturer’s instructions until the fractures occurred. NFC (Table 1) and the length of the broken part (Table 2) were calculated. There was a statistically significant difference between NFC at 4°C (p = 0.00). The highest NFC was observed in the VDW.ROTATE group and the lowest was observed in the HyFlex EDM group. A significant difference was noted between the NFC of each file group at 35°C (p = 0.00). The highest NFC was observed in the HyFlex EDM, and the lowest was observed in the TruNatomy. A significant difference was found between the NFC of each file group at 60°C (p = 0.00). The highest NFC was observed in the VDW.ROTATE, and the lowest was observed in the Trunatomy.

**Table 1:** The number of cycles to fracture of files at different temperatures. Different lowercase letters indicate differences in the same row, and different uppercase letters in the same column (*p < 0.05).

		HyFlex EDM	VDW.ROTATE	TruNatomy	p^1^
4°C	Mean ± SD	4,994.02 ± 851.35	22,839.60 ± 5999.46	9,152.63 ± 1,824.59	<0.001
	Median	5,266.85^aA^	21,124.60^bA^	9,607.25^cA^	
35°C	Mean ± SD	3,288.13 ± 619.97	2,058.67 ± 437.95	823.24 ± 142.90	<0.001
	Median	3,308.95^aB^	1,890.35^bB^	845.80^cB^	
60°C	Mean ± SD	673.52 ± 238.75	889.96 ± 273.35	400.24 ± 96.97	<0.001
	Median	767.00^bC^	944.70^bC^	391.70^aC^	
p^1^		<0.001	<0.001	<0.001	

**Table 2:** The values of the broken part length of the files at different temperatures (mm). Different lowercase letters indicate differences in the same row, and different uppercase letters in the same column (*p < 0.05).

		HyFlex EDM	VDW.ROTATE	TruNatomy	
4°C	Mean ± SD	5.39 ± 2.18	6.81 ± 2.37	3.10 ± 0.76	<0.001
	Median	6.15^bA^	6.25^bA^	2.82^aA^	
35°C	Mean ± SD	7.60 ± 0.33	5.20 ± 1.56	4.36 ± 0.26	<0.001
	Median	7.55^aB^	5.42^bAB^	4.35^bB^	
60°C	Mean ± SD	6.96 ± 1.33	4.67 ± 1.15	4.50 ± 1.36	<0.001
	Median	7.53^aAB^	4.80^bB^	4.56^bB^	
p^1^		0.009	0.017	0.002	

On intragroup comparison, NFC was the highest at 4°C and the lowest at 60°C, with the NFC being statistically significant in all files.

A significant difference was observed between the files in broken part length at all three temperatures (p = 0.00) (Table 2). At 4°C, TruNatomy had a statistically smaller part length than the others, while no significant difference was observed between HyFlex EDM and VDW.ROTATE.

At 35°C and 60°C, HyFlex EDM had statistically larger fracture lengths than the others, while no significant difference was found between VDW.ROTATE and TruNatomy files.

When the in-group comparison results were examined, HyFlex EDM and TruNatomy had shorter fracture lengths at 4°C than the other temperature groups, but no significant difference was found between 35°C and 60°C. There was no significant difference between the temperatures of 35°C, 60°C, and 4°C in the VDW.ROTATE file.

**Figure 1:**
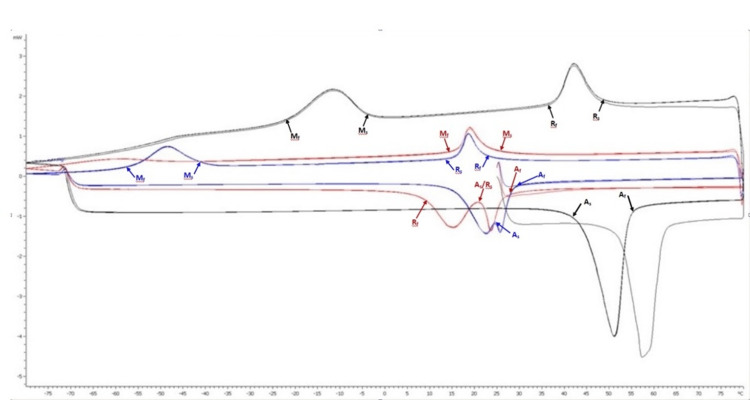
Differential scanning calorimetry charts. Transformation temperature curves of each instrument are identified with different colored lines (black: HyFlex EDM; blue: TruNatomy; red: VDW.ROTATE).

The cyclic fatigue resistance findings of the three rotary file systems in the study were consistent with the martensite/austenite transformation temperatures obtained by DSC analysis (Table 3). The DSC graph of HyFlex EDM shows that it does not contain an austenite phase at 4°C and 35°C, which is the intracanal temperature, and it comprises an austenite phase at 60°C. The DSC graph of VDW.ROTATE shows that at all temperatures above 29°C, the file comprises an austenite phase, while at 4°C, it contains only a martensite phase. The DSC graph of the TruNatomy file shows that, while it does not contain an austenite phase at 4°C, it comprises an austenite phase at temperatures of 35°C and 60°C. The superimposed image of the DSC analyses of the files is shown in Figure 1.

**Table 3:** Phase transformation temperatures of the files measured by differential scanning calorimetry analysis.

	Ms	Mf	Rs	Rf	As	Af
HyFlex EDM	-2.95	-22.56	48.68	37.40	43.65	56.40
VDW.ROTATE	26.17	15.28	21.8	10.48	21.8	29.65
TruNatomy	-40	-57.5	24.3	14.6	26	30

**Figure 2:**
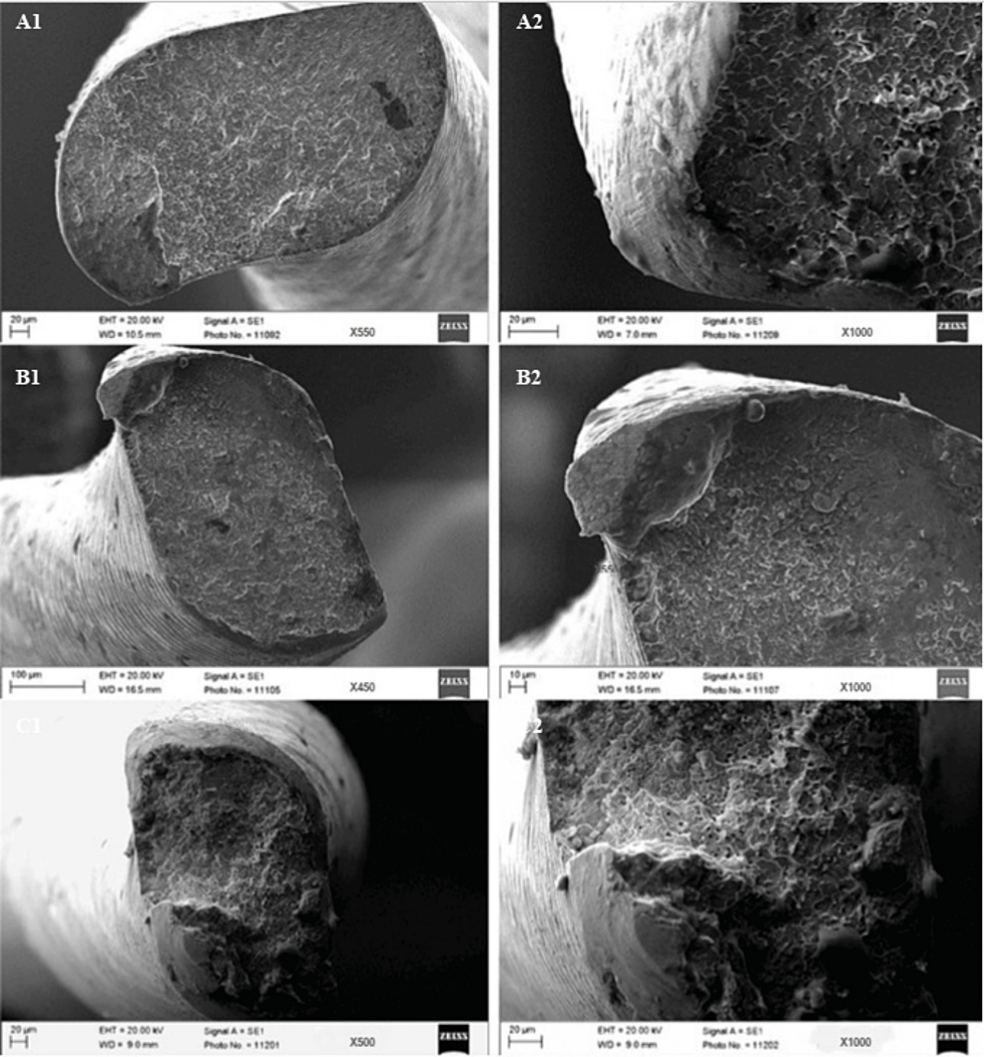
Scanning electron microscopy images of the typical morphology of fractured surface of the VDW.ROTATE at 4°C (A1 magnification 550× and A2 magnification 1,000×), 35°C (B1 magnification 450× and B2 magnification 1,000×), and 60°C (C1 magnification 500× and C2 magnification 1,000×). -

The fracture mechanism was examined using SEM images taken from all files at different magnifications. The typical surface morphology of cyclic fatigues of VDW.ROTATE is shown in Figure 2 at different temperatures. The presence of crack initiation areas, fatigue lines, overloaded areas, micro-voids, and surface pits formed close to the fracture line shows that all file groups were broken because of cyclic fatigue.

## Discussion

Chemomechanical shaping of the canal is one of the important steps of root canal treatment. Fractures in the files used in root canal preparation affect the success of root canal treatment [19]. Most cyclic fatigue studies have been performed under experimental conditions using a static model [9]. Moreover, the cyclic fatigue studies were conducted using a dynamic model with up-down motion [17]. The pecking motion made during root canal shaping in clinical conditions is better simulated by dynamic modeling [20]. Moving the instrument axially prevents the concentration of tensile and compressive stresses at one point during root canal shaping, allowing the distribution of tensile force across a larger portion, increasing the number of fracture cycles [21], and significantly increasing the life of the instrument [22]. The limitation of the study is the evaluation of cyclic fatigue on artificial metal canals, not on the dentin surface, unlike in the clinic. The instruments are used in teeth with different root canal curvatures and different dentin structures, and the instruments are exposed to different stresses in each tooth. However, this difference prevents the standard evaluation of the files. For this reason, artificial metal channels were preferred to set up a standard test setup.

Sodium hypochlorite is the most commonly used irrigation solution during root canal preparation because of its antimicrobial and tissue-dissolving properties [11]. Using heated or cooled solutions can promote the NiTi phase transition of the instrument to a more austenitic or martensitic phase, respectively, which can affect the number of fracture cycles [9]. The phase transitions give the files different properties and increase the cyclic fracture strength. This study aimed to analyze the phase transformation behaviors and evaluate the effect of different temperatures on the fracture strength of the files.

The effect of temperature on cyclic fatigue has been reported in various studies and an increase in NFC has been observed depending on the decrease in temperature [9,12,23]. Cyclic fatigue resistance has been tested at room temperature (25°C) in previous studies, but the intracanal temperature is warmer (35°C) [23]. In our study, room temperature was not included in the groups because the irrigation solutions used in the study are quickly balanced according to body temperature; therefore, it is important to test the cyclic fatigue of files at body temperature rather than room temperature [24]. Conventional superelastic NiTi alloys are in the austenitic phase (hard and strong) at the intracanal temperature, and can be converted to the martensitic phase (soft and ductile) by applying stress and cooling. The increased ductility of a file in the martensite phase makes it highly elastic [10]. In our study, files broke faster at 60°C compared to other temperatures, and according to DSC analysis, all files were found to be in the austenite phase at 60°C. In a similar study, it was found that the files showed the lowest NFC at high temperatures [25]. At a temperature higher than the transformation temperature, the NiTi alloy is mainly composed of austenite, and the phase imparts high hardness and low flexibility with the instrument [26]. This is an important reason for the early breakage of files.

Another reason for fatigue resistance is that the nature of the martensitic phase transformation has damping properties that make crack propagation more difficult because of the large number of interfaces observed. These interfaces also improve resistance to fatigue crack growth, providing higher resistance to cyclic fatigue [10]. This may be another reason for the large cyclic fatigue resistance difference observed in our study at 4°C compared to other temperatures.

HyFlex EDM showed the highest NFC at intracanal temperature (35°C), while TruNatomy showed the lowest NFC. In studies conducted at body temperature, TruNatomy showed lower NFC than VDW.ROTATE and HyFlex CM [13,27].

The highest NFC at 60°C was seen for VDW.ROTATE and the lowest for TruNatomy. The cross-sectional shape may have caused this significant difference at this temperature, where all files were in the same phase (austenite). Although all three files were specially heat-treated files that were produced recently, there are few studies, especially with VDW.ROTATE. In a previous study [27], high cyclic fatigue resistance of this file was observed in line with our results. VDW.ROTATE may have shown high fatigue resistance with its S-shaped cross-section. Grande et al. [28] reported that the metal volume at the area of maximum stress area contributes to the fatigue resistance of the files. Mtwo files (VDW, Munich, Germany) with an S-shaped cross-section design and lower metal volume presented higher fatigue resistance than ProTaper files (Dentsply Sirona, Ballaigue, Switzerland) with the triangular design and larger metal mass [28]. In addition, according to this study, files can change with factors affecting the cross-sectional area, such as core diameters and flute depths [28]. Several studies have concluded that files produced with the S-shaped cross-section design can increase fatigue resistance because of the reduction in metal volume at the point of maximum curvature [29]. In addition, the design features, dimensions [30], and the file diameter at the maximum curvature point of the canal [31] are important factors affecting cyclic fatigue, as well as the alloys from which the files are made. VDW.ROTATE 25.04, TruNatomy 26.04, and HyFlex EDM had 25 tip diameters and variable taper from 08 to 04. That the files have different tip diameters and tapers may also have caused cyclic fatigue to be affected by these factors, which was the second limitation of our study.

In our study, significant differences were observed between the lengths of the broken parts. The HyFlex EDM had a greater length of value at all temperatures. TruNatomy and VDW.ROTATE had similar results overall. In various studies [5,25], the length of the parts did not make a significant difference, which was explained because the maximum stress point was the same in all cases because of the static cyclic fatigue mechanism. However, the dynamic system caused the stress point to not always be constant. In a study [14], Reciproc Blue and VDW.ROTATE were evaluated in terms of cyclic fatigue and fracture length. The fracture length was significantly larger for Reciproc Blue. As a result, the differences in the design and alloys of the files can change the positions of the maximum stress points.

In our study, SEM images were taken for each group of files broken in the test setup, and the images were examined fractographically. The purpose of the fractographic examination was to identify features on the fractured surface that showed the source and direction of the crack, causing the material to break [32]. Some marks on the fracture surface indicate the actual mechanism of the fracture process [4]. Two mechanisms, cyclic fatigue and torsional fatigue fracture, play a role in the fracture of NiTi rotary files. Cyclic fatigue is characterized by fatigue lines. Torsional fracture exhibits circular wear marks on the fractured surface. In the images, fatigue lines because of cyclic fatigue and crack initiation lines were clear. Because of the fractographic analysis of the broken files in the study, it was determined that all files were broken by cyclic fatigue.

In general, flexible files show less resistance to torsional loads and more resistance to cyclic fatigue than more rigid files [33]. In our study, only the effect of ambient temperature on cyclic fatigue was examined, and it was found that a cold environment significantly increased cyclic fatigue resistance. However, the effect of ambient temperature change on torsional fatigue and cutting efficiency of the file should be investigated in future studies.

## Conclusions

Breakage of endodontic files affects the success of root canal treatment. The resistance to cyclic fatigue of all files decreased significantly as the temperature increased. Further studies to find a strategy that could prolong the usage life of endodontic files are recommended in clinical practice to design a mechanism/device for directly cooling the intracanal or irrigation solutions.
